# Chronic Repression of mTOR Complex 2 Induces Changes in the Gut Microbiota of Diet-induced Obese Mice

**DOI:** 10.1038/srep30887

**Published:** 2016-07-29

**Authors:** Mi-Ja Jung, Jina Lee, Na-Ri Shin, Min-Soo Kim, Dong-Wook Hyun, Ji-Hyun Yun, Pil Soo Kim, Tae Woong Whon, Jin-Woo Bae

**Affiliations:** 1 Department of Life and Nanopharmaceutical Sciences and Department of Biology, Kyung Hee University, Seoul 130-701, Korea

## Abstract

Alterations in the gut microbiota play a crucial role in host physiology and metabolism; however, the molecular pathways underlying these changes in diet-induced obesity are unclear. Mechanistic target of rapamycin (mTOR) signaling pathway is associated with metabolic disorders such as obesity and type 2 diabetes (T2D). Therefore, we examined whether changes in the regulation of mTOR signaling induced by diet (a high-fat diet [HFD] or normal-chow diet) and/or therapeutics (resveratrol [a specific inhibitor of mTOR complex 1] or rapamycin [an inhibitor of both mTOR complex 1 and 2]) altered the composition of the gut microbiota in mice. Oral administration of resveratrol prevented glucose intolerance and fat accumulation in HFD-fed mice, whereas rapamycin significantly impaired glucose tolerance and exacerbated intestinal inflammation. The abundance of *Lactococcus*, *Clostridium* XI, *Oscillibacter*, and *Hydrogenoanaerobacterium* increased under the HFD condition; however, the abundance of these species declined after resveratrol treatment. Conversely, the abundance of unclassified *Marinilabiliaceae* and *Turicibacter* decreased in response to a HFD or rapamycin. Taken together, these results demonstrated that changes in the composition of intestinal microbiota induced by changes in mTOR activity correlate with obese and diabetic phenotypes.

Obesity is a major risk factor for various chronic diseases, including type 2 diabetes (T2D), cardiovascular disease, hypertension, non-alcoholic fatty liver disease and cancer^1^. The fundamental cause of obesity is an imbalance between energy intake from foods and energy expenditure through basal metabolism, physical activity and thermogenesis^2^. Since the basal metabolism rate (BMR) accounts for about 60–75% of the total energy expenditure^2^, a low BMR per unit of body weight is one of the risk factors for obesity^3^. Moreover, energy balance is influenced by complex interactions between genetic, environmental and psychosocial factors^4^. With respect to energy intake, changes in gastrointestinal (GI) motility contribute to obesity by regulating not only the digestive efficiency but also appetite and satiety^5^. Interestingly, recent studies suggest that gut microbiota play an important role in energy harvest and obesity via interactions with GI motility^6^,^7^.

The composition of the gut microbiota is influenced by the genetic background, immune status, age, sex and (especially) diet of the host^8^. Although a high-fat diet (HFD) alters the composition of the intestinal microbiota^9^, recent studies show that the gut microbiota themselves promote obesity and a diabetic phenotype^10^,^11^. By contrast, several species of intestinal microbe have a beneficial effect on obesity and obesity-related metabolic disorders via their ability to modulate immune homeostasis^12^,^13^. We recently demonstrated that oral administration of the mucin-degrading bacterium *Akkermansia muciniphila*, which is markedly more abundant in metformin-treated HFD-fed mice than in HFD-fed control mice, improved glucose tolerance and alleviated adipose tissue inflammation in diet-induced obese (DIO) and diabetic mice by inducing proliferation of mucin-producing intestinal goblet cells and adipose tissue-resident Foxp3+ regulatory T cells^14^. Also, previous studies revealed that compositional changes in the gut microbiota resulting from genetic ablation of antimicrobial peptides such as defensin and RegIII-gamma could affect host physiology^15^,^16^. Even though studies have shown consistently that gut microbiota regulate host metabolism and immune status, both the molecular mechanisms by which energy-rich diets alter the gut microbiota, and the interactions between the host and the gut microbiota that maintain metabolic homeostasis, remain unclear.

Mechanistic target of rapamycin (mTOR) is a central regulator of energy storage and consumption, and is implicated in deleterious states such as cancer, metabolic diseases and ageing^17^. Because over-activation of mTOR complex 1 (mTORC1) signaling by excessive energy intake plays a crucial role in metabolic disorders^17^, mTOR inhibitors such as rapamycin (23,27-epoxy-3H-pyrido[2,1-c][1,4]oxaazacyclohentriacontine), metformin (1,1-dimethylbiguanide hydrochloride) and resveratrol (3,4′,5-trihydroxy-trans-stilbene) are used to treat those suffering from obesity and T2D^14^,^18^,^19^. Both metformin and resveratrol activate AMP-activated protein kinase (AMPK), which in turn inhibits mTORC1^14^,^18^. Although rapamycin is also thought to inhibit mTORC1-S6 kinase 1 (S6K1) activation, long-term treatment with rapamycin also inactivates mTOR complex 2 (mTORC2) and Akt (Ser473), leading to impaired glucose homeostasis and insulin activity^19^. Several studies have shown increased activation of AMPK in the liver, muscle and colon tissue of germ-free mice that are resistant to HFD-induced obesity, suggesting that the intestinal microbiota may modulate AMPK activation in the host^20^,^21^. These findings raise the possibility that changes in mTOR signaling may have both direct and indirect effects on the intestinal microbiota, which in turn would contribute to obese and diabetic phenotypes.

Here, we examined the effects of resveratrol and rapamycin on mTOR signaling, metabolic marker expression, and the composition of the gut microbiota in both normal and DIO mice. The results show that resveratrol and rapamycin have marked effects not only on the mTOR signaling activity and metabolic marker expression but also on the composition of gut microbiota, suggesting that specific microbial groups correlate with the pathophysiological phenotypes associated with obesity and T2D.

## Results

### Resveratrol, but not rapamycin, mitigates HFD-induced obesity

**Figure 1:**
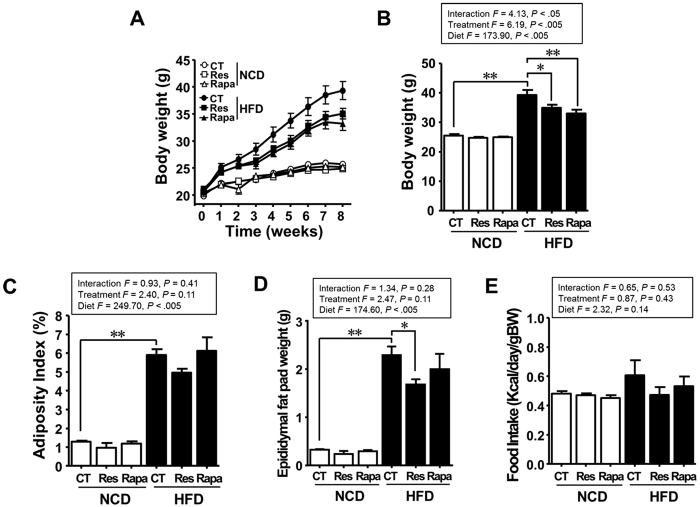
Resveratrol, but not rapamycin, mitigates HFD-induced obesity. (**A**) Effects of resveratrol or rapamycin treatment on temporal changes in the body weight (BW) of NCD- or HFD-fed mice over 8 weeks. (**B**) BW, (**C**) adiposity index (AI), and (**D**) epididymal fat pad weight measured after 8 weeks of resveratrol or rapamycin treatment (**E**) Food intake (FI) by the resveratrol- or rapamycin-treated groups. Data are expressed as the mean ± SEM (n = 5 per group). *F*- and *p*-values are from two-way ANOVA after Bonferroni’s post hoc test. ^*^P < 0.05, ^**^P < 0.005.

As expected, the body weight (BW) and fat deposition of HFD-fed mice were higher than those of normal-chow diet (NCD)-fed mice (P < 0.005; Fig. 1). The BW and epididymal fat pad weight of the HFD-fed mice treated with resveratrol (HFD-Res) were significantly lower than those of the HFD-fed mice without any additional treatment (HFD-CT) (P < 0.05; Fig. 1A–C). By contrast, epididymal fat pad weight in the HFD-fed mice treated with rapamycin (HFD-Rapa) was not lower than that in the HFD-CT mice (P = 0.60), although BW was markedly lower than that in the HFD-Res mice (P < 0.005). There were no marked differences in the amount of food consumed by the animals in the control and treatment groups (Fig. 1E). Thus, resveratrol mitigated both BW gain and fat deposition. However, although rapamycin mitigated weight gain in HFD-fed mice, it had no effect on fat mass.

### Resveratrol improves, whereas rapamycin impairs, glucose homeostasis

**Figure 2:**
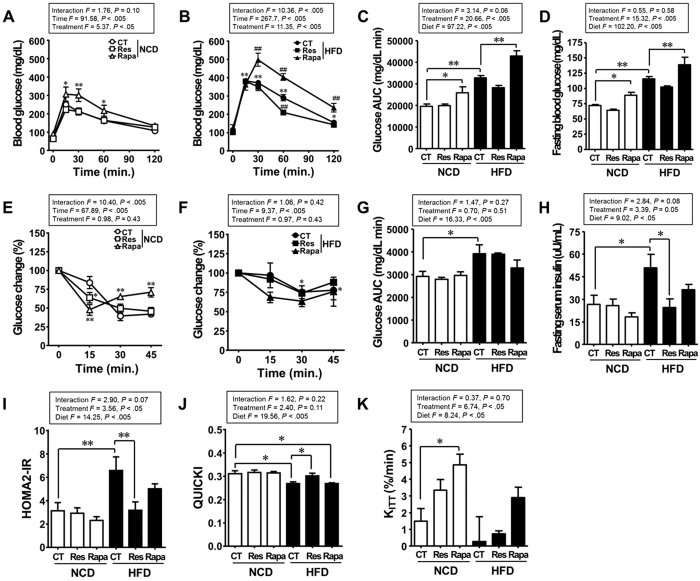
Resveratrol improves, whereas rapamycin impairs, glucose homeostasis. (**A,B**) Blood glucose levels (repeated measures two-way ANOVA after Bonferroni’s post hoc test, *F* = 13.88; *P* < 0.005 for interaction, *F* = 186.0; *P* < 0.005 for time, *F* = 92.14; *P* < 0.005 for diet, ^*^P < 0.05; ^**^P < 0.005 compared with NCD-CT, ^#^*P* < 0.05; ^##^*P* < 0.005 compared with HFD-CT) and (**C**) area under the curve (AUC) during the glucose tolerance test (GTT) (n = 5 per group). (**D**) Fasting blood glucose levels (n = 10 per group). (**E,F**) Blood glucose levels (repeated measures two-way ANOVA after Bonferroni’s post hoc test, *F* = 3.33; *P* = 0.06 for interaction, *F* = 19.65; *P* < 0.005 for time, *F* = 5.63; *P* = 0.08 for diet, ^*^P < 0.05; ^**^P < 0.005 compared with NCD-CT) and (**G**) AUC during the insulin tolerance test (ITT) (n = 3 per group). (**H**) Fasting serum insulin levels (n = 5 per group). (**I**) HOMA2 indices and (**J**) QUICKI were calculated from fasting glucose and insulin levels (n = 5 per group). (**K**) The rate constant for plasma glucose disappearance (K_ITT_) during the insulin tolerance test (ITT) (n = 3 per group). Mice were overnight-fasted (16 h) before the GTT and ITT. Data are expressed as the mean ± SEM. *F*- and *p*-values are from two-way ANOVA after Bonferroni’s post hoc test. ^*^P < 0.05, ^**^P < 0.005.

To determine whether resveratrol or rapamycin affect glucose homeostasis, we performed the glucose tolerance test (GTT) and insulin tolerance test (ITT) at the end of the treatment period. As expected, compared with the NCD-fed mice without any additional treatment (NCD-CT), the HFD-CT group showed an increase in the area under the curve (AUC) during GTT and ITT, along with an increase in fasting blood glucose and fasting serum insulin levels (Fig. 2A–H). The homeostatic model assessment 2 (HOMA2) model and quantitative insulin sensitivity check index (QUICKI) are measures of insulin resistance and insulin sensitivity, respectively, and are derived from fasting glucose and insulin levels^22^,^23^. Here, we found that the HOMA2-insulin resistance (IR) index was significantly higher and QUICKI significantly lower in the HFD-CT group than in the NCD-CT group (Fig. 2I,J). These results indicate that a HFD impairs glucose tolerance and induces insulin resistance. When compared with the HFD-CT group, the HFD-Res group showed significantly improved glucose intolerance and insulin resistance, both of which were confirmed by a reduction in the blood glucose level at 60 min. during GTT, reduced levels of fasting serum insulin, a lower HOMA2-IR index and a higher QUICKI index (Fig. 2B,H–J). Unexpectedly, chronic rapamycin treatment led to impaired glucose homeostasis, including in the NCD-fed mice. There was a marked increase in glucose intolerance (AUC of the GTT) and fasting blood glucose level in the rapamycin-treated mice compared with the control mice (Fig. 2A–D). However, the rate constant for plasma glucose disappearance (K_ITT_) during ITT was higher in the NCD-fed mice treated with rapamycin (NCD-Rapa) than in the NCD-CT mice (Fig. 2K). Taken together, these results suggest that chronic rapamycin treatment is associated with impaired insulin synthesis or secretion, but not with insulin resistance. Thus, resveratrol treatment prevented hyperinsulinaemia and insulin resistance in DIO mice, whereas rapamycin triggered glucose intolerance and disrupted pancreatic beta cell function in both healthy and DIO mice.

### Resveratrol ameliorates, whereas rapamycin exacerbates, intestinal inflammation

We next examined the effects of resveratrol and rapamycin on gut inflammation in NCD or HFD-fed mice by measuring faecal lipocalin-2 (LCN-2), a sensitive biomarkers for low-grade intestinal inflammation^24^, and colon length. The concentration of faecal LCN-2 in HFD-CT mice was significantly higher than that in NCD-CT mice (P < 0.05; Supplementary Fig. S2A). Moreover, higher levels of LCN-2 were observed in HFD-Rapa mice than in HFD-CT mice (P < 0.05). Furthermore, the length of the colon in HFD-Rapa mice was significantly shorter than that in NCD-CT mice (P < 0.05; Supplementary Fig. S2B), and the levels of faecal LCN-2 correlated negatively with colon length (*r* = −0.92; P < 0.005) (Supplementary Fig. S2C). These observations indicate that rapamycin aggravates intestinal inflammation in DIO mice.

### Resveratrol specifically inhibits mTORC1, whereas rapamycin inhibits both mTORC1 and 2

The ratio of phosphorylated S6 (Ser235/236) to total S6 protein (p-S6/total S6) was significantly higher, and the ratio of phosphorylated Akt1 (Ser473) to total Akt1 (p-Akt1/total Akt1) lower, in HFD-CT mice than in NCD-CT mice (Supplementary Fig. S3A,B). These results suggest that a HFD activates mTORC1. By contrast, the p-S6/total S6 ratio was lower, and the p-Akt1/total Akt1 ratio and cAMP level were higher, in HFD-Res mice than in HFD-CT mice (Supplementary Fig. S3C), indicating that resveratrol inhibits mTORC1 and activates mTORC2 in HFD-fed mice. By contrast, chronic rapamycin treatment led to reduced p-S6/total S6 and p-Akt1/total Akt1 ratios, regardless of diet, demonstrating that rapamycin suppresses the activity of both mTORC1 and 2.

### Effect of resveratrol and rapamycin on the gut microbiota

To ascertain the effects of differential regulation of mTOR activity on the gut microbiota, we analysed the sequences of bacterial 16S rRNA gene amplicons using 454 pyrosequencing technology. After quality control processing, we obtained 215,932 high-quality sequences, with an average of 7,198 (±358) reads per sample. Compared with NCD-CT, we observed a significant reduction in the operational taxonomic units (OTUs) and bacterial alpha diversity indices in the HFD-fed groups (Supplementary Table S1). However, there was no significant difference in the alpha diversity indices between resveratrol- and rapamycin-treated groups. Although the relative abundance of *Firmicutes* (*P* < 0.005) was higher, and that of *Bacteroidetes* lower (*P* < 0.005), in HFD-CT mice than in NCD-CT mice, resveratrol and rapamycin treatment under NCD or HFD feeding conditions did not significantly affect the faecal *Firmicutes*/*Bacteroidetes* (F/B) ratio (Supplementary Fig. S4).

**Figure 3:**
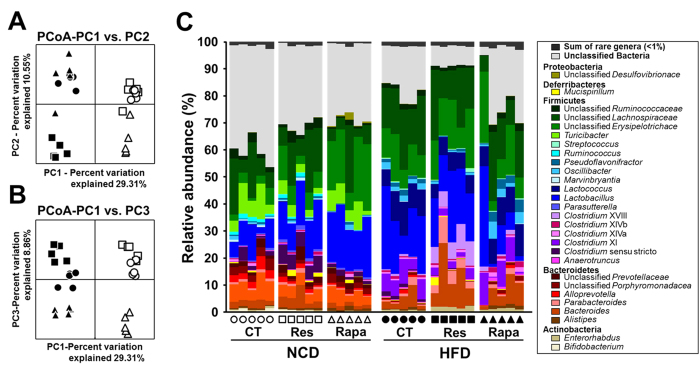
Changes in the faecal bacterial community following resveratrol or rapamycin treatment. Bacterial communities were clustered using unweighted UniFrac distance-based principal coordinates analysis (PCoA). (**A**) Principal coordinate (PC) 1 versus PC2 and (**B**) PC1 versus PC3. The percentage variation in the plotted PC is indicated on the axes. (**C**) Bar charts showing the relative abundance (%) of different bacterial genera in the different diet and treatment groups. Each group of mice is represented by a different symbol or bar on the x axis of the graph, and each spot or column indicates one sample (n = 5 per group).

Using principle coordinate analysis (PCoA) based on unweighted UniFrac distances, we next compared the composition of the gut microbiota in the diet and treatment groups. The PC1 axis of the PCoA clearly separated the gut bacterial community according to dietary type (Fig. 3A). Furthermore, each resveratrol- or rapamycin-treated group formed a distinct cluster from the control groups along the PC3 axis (Fig. 3B), suggesting that resveratrol or rapamycin has differential effects on gut microbial communities in NCD- and HFD-fed mice.

**Figure 4:**
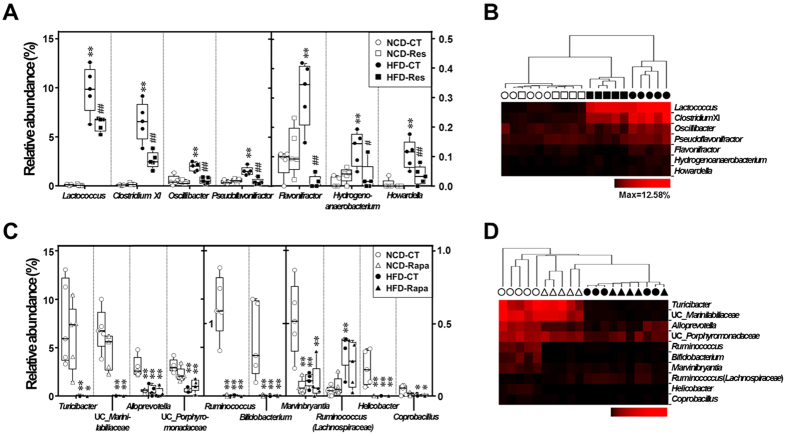
Marked differences in the relative abundance of gut bacterial genera in the different diet and treatment groups. Effect of (**A,B**) resveratrol or (**C,D**) rapamycin on the relative abundance of 7 or 10 bacterial genera, and hierarchical clustering analysis of these bacterial profiles based on the Manhattan distance, were examined in NCD- and HFD-fed mice. Data are expressed as the mean ± SEM (n = 5 per groups). *F*- and *p*-values are from two-way ANOVA after Bonferroni’s post hoc test (Supplementary Table S2). ^*^P < 0.05 and ^**^P < 0.005 compared with NCD-CT. ^#^*P* < 0.05 and ^##^*P* < 0.005 compared with HFD-CT.

To determine whether resveratrol or rapamycin induce more specific changes in the gut bacterial taxa, we performed a nearest shrunken centroid (NSC) analysis. Statistical analysis of variance (ANOVA) and NSC analyses revealed that changes in the abundance of 17 taxa accounted for the observed changes in the gut microbiota induced by diet and resveratrol or rapamycin treatment, which suggests a correlation between the antidiabetic effect of resveratrol or diabetic effect of rapamycin and specific subsets of gut bacteria. The relative abundances of *Lactococcus*, *Clostridium* XI, *Oscillibacter*, *Pseudoflavonifractor*, *Flavonifractor*, *Hydrogenoanaerobacterium* and *Howardella* were significantly higher in HFD-fed mice, and resveratrol treatment reversed these HFD-induced changes in bacterial abundance (Figs 3C and 4A). Furthermore, hierarchical clustering showed that the bacterial profiles of HFD-Res mice resembled more those of NCD-fed mice than those of HFD-CT mice (Fig. 4B). By contrast, rapamycin changed the relative abundances of *Turicibacter*, unclassified *Marinilabiliaceae*, *Alloprevotella*, unclassified *Porphyromonadaceae*, *Ruminococcus*, *Bifidobacterium*, *Marvinbryantia*, *Ruminococcus* (*Lachnospiraceae*), *Helicobacter*, and *Coprobacillus* to those observed in HFD-fed mice (Figs 3C and 4C). With the exception of *Ruminococcus* (*Lachnospiraceae*), these bacteria were more abundant in NCD-fed mice than in HFD-fed or rapamycin-treated mice. The results were confirmed by hierarchical clustering analysis, which revealed that the bacterial profiles in NCD-Rapa mice clustered more closely with those in HFD-fed mice than with those in NCD-CT mice (Fig. 4d). Thus, resveratrol prevented changes in the relative abundance of specific HFD-induced bacteria, resulting in bacterial levels similar to those seen in NCD-fed mice, while rapamycin contributed to HFD-induced changes in gut microbiota.

**Figure 5:**
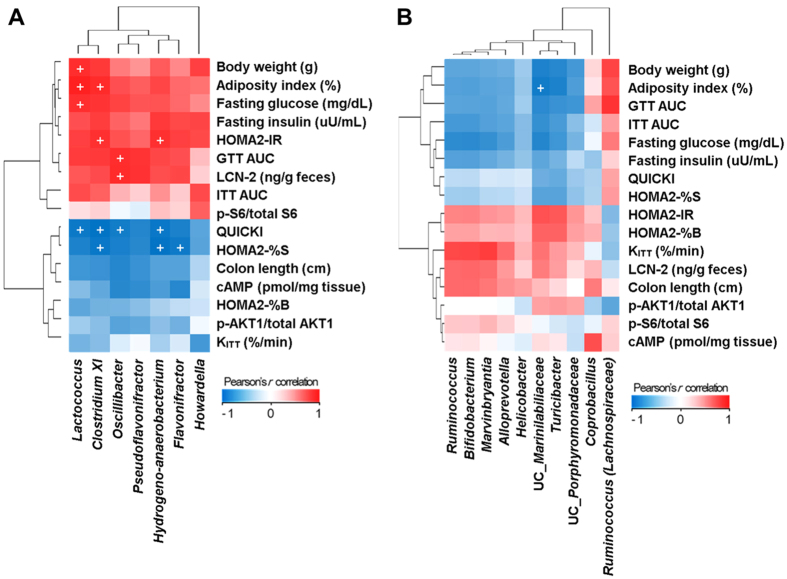
Pearson’s *r* correlation coefficients heat maps showing the association between metabolic markers and the abundance of specific bacterial genera after (**A**) resveratrol or (**B**) rapamycin treatment. Given the large number of correlation tests performed, a significance threshold of *P* < 0.005 was used, which is indicated by ‘+’.

With this in mind, we performed linear regression analysis to examine for a possible connection between the abundance of specific gut bacteria and host metabolic parameters. Despite the small sample size, Pearson’s correlation analysis showed that several metabolic parameters correlated with the abundance of specific populations of gut bacteria (Fig. 5). Specifically, the relative abundances of five taxa (*Lactococcus*, *Clostridium* XI, *Oscillibacter*, *Hydrogenoanaerobacterium* and *Flavonifractor*) were higher in HFD-fed mice and were reduced by resveratrol treatment. These abundances correlated positively with biomarkers for metabolic syndrome (e.g., BW, AI, AUC during GTT, fasting blood glucose level, the HOMA2-IR index, and faecal LCN-2 levels), and correlated negatively with biomarkers for insulin sensitivity (e.g., QUICKI and HOMA2-%S indices). Conversly, the relative abundance of unclassified *Marinilabiliaceae* which were lower in HFD-fed and rapamycin-treated mice, correlated negatively with AI. Therefore, HFD and rapamycin not only contribute to the mTOR signaling activity and the host diabetic phenotype, but also influence the composition of the gut microbiota.

## Discussion

We previously showed that oral administration of mucin-degrading *Akkermansia muciniphila*, which is markedly more abundant in metformin-treated HFD-fed mice, resulted in improved glucose homeostasis and reduced adipose tissue inflammation via its ability to induce goblet cells in the intestine and regulatory T cells in adipose tissue^14^. Since metformin is a key regulator of mTOR signaling, the present study examined whether the mechanisms underlying host central energy metabolism, which are controlled by differential regulation of the mTOR pathway, involve changes in the composition of gut microbiota.

Consistent with the results of a previous study^18^, we found that resveratrol protected HFD-fed mice against glucose intolerance, hyperinsulinaemia, fat deposition and BW gain. These effects seem to be attributed to the various mechanisms of resveratrol, including the inhibition of mTORC1 signaling pathway. Conversely, prolonged rapamycin treatment disrupted glucose homeostasis and pancreatic beta cell function in both NCD and HFD-fed mice by inhibiting mTORC1 and mTORC2 and preventing Akt activation^19^. Consistent with our results, recent studies indicate that mTOR signaling is important not only for glucose and lipid metabolism^25^ but also for the regulation of gut barrier function^26^ and immune homeostasis via its effects on immune cell profiles^27^ and cytokine production^28^, both of which may play an essential role in controlling gut microbiota and affect the pathogenesis of obesity and diabetes. In addition, over-activation of mTORC1 signaling via TSC2 inactivation suppresses the differentiation of intestinal goblet and Paneth cells^29^, both of which might contribute to dysbiosis of gut microbiota by reducing the production of mucus and antimicrobial peptides, respectively^16^,^30^. Interestingly, chronic rapamycin treatment has been suggested to aggravate intestinal inflammation in diet-induced obese mice. Recent various studies have confirmed that rapamycin treatment contributes to inflammation by promoting the expression of pro-inflammatory cytokines, such as interleukin-12 (IL-12), IL-6, IL-1β and tumor necrosis factor-α, and by inhibiting of IL-10 expression in immune cells^31^,^32^,^33^. These pro-inflammatory responses have been attributed to the activation of the nuclear factor-kB (NF-kB) and/or forkhead box O1 (FoxO1) or the inhibition of signal transducer and activator of transcription 3 (STAT3). In particular, mTORC2 signaling is crucial for regulating immune status because inactivity of Akt (a downstream target of mTORC2 signaling) leads to impairment of FoxO1 phosphorylation^34^. Impairment of FoxO1 phosphorylation promotes not only gluconeogenesis in the liver, protein catabolism in muscle, and apoptosis in pancreatic beta cells, but also inflammation of adipose tissue by increasing TLR4-mediated signaling in mature macrophages^34^. Macrophages in adipose tissue play an important role in the pathogenesis of insulin resistance and obesity via their ability to produce pro-inflammatory cytokines^35^. Furthermore, FoxO1 directly regulates the production of antimicrobial peptides by binding to the regulatory region of the antimicrobial peptide gene promoter^34^, thereby modulating the composition of the gut microbial community^16^. Here, we showed that the prolonged rapamycin treatment is associated with obese and diabetic phenotypes, whereas resveratrol treatment is associated with improved metabolic biomarker profiles in DIO mice.

We also found that the pathophysiology induced by changes in the regulation of the mTOR pathway were associated with a marked shift in the composition of gut microbiota. When considering the recent studies showing that dysbiosis of the gut microbiota directly affects host metabolism^36^,^37^, the changes in gut microbiota by resveratrol or rapamycin treatment may have the potential to affect DIO and diabetes. We also found that the relative abundances of 17 bacterial taxa were changed significantly after resveratrol or rapamycin treatment. Among these, *Lactococcus*, *Clostridium* XI, *Oscillibacter*, and *Hydrogenoanaerobacterium* were most strongly associated with obese and diabetic phenotypes. The proportions of these taxa correlated significantly and positively with BW, AI, glucose intolerance, insulin resistance and intestinal inflammatory marker gene expression. Several studies have highlighted a link between the abundance of these taxa and HFD-induced obesity^14^,^38^,^39^; however, little is known about their physiological roles. Qiao *et al*.^38^ showed that an increase in the *Lactococcus* population in Peyer’s patches is associated with an obesity-prone phenotype, and was positively correlated with the levels of pro-inflammatory cytokines such as interleukin (IL)-6 and TNF-α, but negatively associated with the levels of anti-inflammatory cytokines such as IL-10. Moreover, mice fed a NCD or a HFD with 30% caloric-restriction showed a significant reduction in the abundance of *Lactococcus*^40^. Our recent study also demonstrated that HFD-fed mice orally administered with metformin had lower abundances of *Lactococcus* and *Hydrogenoanaerobacterium*^14^. Yoshimoto *et al*.^41^ showed that the proportion of *Clostridium* XI is markedly higher in HFD-fed mice and is associated with higher levels of deoxycolic acid, a known carcinogen of the colon and liver^41^,^42^. *Oscillibacter* is positively correlated with gut permeability^43^, which can influence adiposity and systemic inflammation in obese prone donors and their GF recipients fed a HF diet^39^. In addition, *Methanobrevibacter smithii*, the predominant microbe in the obese human gut^44^, utilizes hydrogen for methanogenesis and increases energy uptake efficiency by interacting with hydrogen-producing *Hydrogenoanaerobacterium*^45^. Based on these results, we suggest that activating the mTORC2 signaling pathway followed by mTORC1 inhibition with resveratrol suppresses the growth of obesity-associated gut microbiota, such as *Lactococcus*, *Clostridium* XI, *Oscillibacter*, and *Hydrogenoanaerobacterium*.

In conclusion, the results presented herein demonstrate that changes in the composition of the gut microbiota caused by treatment of resveratrol and rapamycin are correlated with alterations in BW, fat deposition, insulin resistance, and intestinal inflammation in DIO mice. Although the identities of specific HFD-related molecules that enrich or diminish certain populations of gut microbes remain to be identified, mTOR signaling would appear to be a key component of the regulation of the composition of gut microbiota in DIO mice.

## Methods

### Animals

Four-week-old male C57BL/6J mice were purchased from Japan SLC, Inc. (Haruno Production Facility, Japan) and maintained in groups of no more than five mice per cage at the animal facility at Kyung Hee University. Mice were housed under specific pathogen-free conditions at 48 ± 6% relative humidity and temperature- and light-controlled conditions (25 ± 1 °C; 14 hr light/10 hr dark cycle) with free access to food and water. After 1 week of acclimation, mice were fed either a NCD (12.41% kcal from fat, 24.52% kcal from protein, 63.07% kcal from carbohydrate; #38057; Purina Korea, Inc., Seoul, South Korea; Supplementary Table S3) or a HFD (60% kcal from fat, 20% kcal from protein, 20% kcal from carbohydrate; #D12492; Research Diets Inc., New Brunswick, USA; Supplementary Table S4). During the 8 week study, male C57BL/6J mice were divided into six groups (5-week-old; n = 5 per group) as follows: (1) an NCD without any additional treatment (NCD-CT); (2) an NCD plus resveratrol (NCD-Res); (3) an NCD plus rapamycin (NCD-Rapa); (4) a HFD without any additional treatment (HFD-CT); (5) a HFD plus resveratrol (HFD-Res); or (6) a HFD plus rapamycin (HFD-Rapa). The resveratrol-treated (NCD-Res and HFD-Res) mice received 200 mg/kg/day of resveratrol (ChromaDex, Inc., Irvine, CA) and the rapamycin-treated (NCD-Rapa and HFD-Rapa) mice received 3 mg/kg/day of rapamycin (Enzo Life Sciences, Inc., Farmingdale, NY) by oral gavage (ethanol-dissolved stock solution diluted in phosphate buffered saline) throughout the experimental period (5 days per week). Control mice were gavaged with phosphate buffered saline alone by the same person. Food intake was manually monitored per cage during a four-week period. All animal experiments were approved by and performed in accordance with the guideline of the committee for care and use of laboratory animals of College of Pharmacy, Kyung Hee University (KHP-2013-08-2-R1).

### Samples collection

Faecal samples were freshly collected after 8 weeks of resveratrol or rapamycin treatment, and then stored at **−**80 °C. At the end of the treatment period, the mice were anesthetized using isoflurane (2-chloro-2-(difluoromethoxy)-1,1,1-trifluoro-ethane) after overnight-fasting (16 hr). Livers, intestines, epididymal fat pads and blood samples were rapidly collected, washed briefly in PBS and stored at **−**80 °C until processing. Epididymal fat pads were weighted for the calculation of the AI (g epididymal fat pads weight/g BW·100). Serum was separated from blood using Microtrainer™ tubes (BD, Franklin Lakes, NJ) for insulin analyses.

### Analysis of glucose homeostasis

A GTT or ITT was performed at the end of the treatment period. Overnight-fasted mice received a glucose load (1.5 g/kg BW) by oral gavage or an insulin load (0.75 U/kg BW) by intraperitoneal injection. A blood glucometer (Accu-Check Performa, Roche) was used to measure blood glucose levels both before and after glucose or insulin loading. The updated homeostatic model assessment (HOMA2), which includes HOMA2-IR (insulin resistance), HOMA2-%B (pancreatic beta cell function) and HOMA2-%S (insulin sensitivity) indices, was used to calculate QUICKI and K_ITT_ as described previously^23^,^46^,^47^. The HOMA2 model was calculated using fasting glucose and fasting insulin levels measured before sacrifice and before glucose loading for the GTT.

### Enzyme-linked immunosorbent assay

To evaluate activation of both mTORC1 and 2 signaling complexes, liver samples were examined using a Mouse/Rat cAMP Parameter Assay Kit (R&D Systems Inc., Minneapolis, MN) for cellular cAMP level and PathScan sandwich ELISA Kits (Cell Signaling Technology, Beverly, MA) for total S6, phosphorylated S6 (Ser235/236), and total Akt and phosphorylated Akt1 (Ser473). To examine Akt1 phosphorylation, 0.5 U of insulin per kg body weight were injected 10 minutes before blood collection. Serum insulin concentrations were measured using Mouse Insulin ELISA kit AKRIN-011T (Shibayagi, Gunma, Japan). To assess intestinal inflammation, the level of faecal LCN-2 was measured using a Mouse Lipocalin-2/NGAL Quantikine ELISA Kit (R&D Systems Inc., Minneapolis, MN) as described by Chassaing *et al*.^24^. All ELISAs were performed according to the manufacturers’ protocols.

### DNA extraction, bacterial 16S rRNA gene amplification and 454 pyrosequencing

Metagenomic DNA was extracted from faecal samples (0.02 g per sample) using the repeated bead beating plus column method as previously described^14^. The V1 and V2 hyper-variable regions of the bacterial 16S rRNA gene were amplified from each extracted DNA sample using barcode primers^14^. Five replicated PCR products per sample were pooled and purified using a QIAquick PCR purification kit (Qiagen, Valencia, CA). The pooled DNA was sequenced using 454 pyrosequencing GS FLX Titanium (Roche 454 Life Sciences, Branford, CT). Sequencing was performed by Macrogen (Seoul, Korea).

### Analysis of bacterial 16S rRNA gene sequences and community comparison

Initial processing of sequence data, quality control, phylotype binning, and taxonomic alignment of raw sequencing reads were performed using Quantitative Insight into Microbial Ecology (QIIME) software (package 1. 8. 0)^48^. The following parameters were used for quality filtering: minimum/maximum length = 200/1000; no ambiguous bases; no primer mismatches; average quality score >25; and homopolymer run <6 nucleotides. The reverse primers were trimmed away from denoised sequences analysed using the Denoiser algorithm^49^. OTUs with 97% sequence identity were clustered using UCLUST software and the Greengenes core set as a reference sequence database^50^. A representative sequence was chosen for each phylotype and aligned against the Greengenes core set using PyNAST^48^. ChimeraSlayer was then used to remove potentially chimeric sequences from the aligned representative sequences^51^. Taxonomic classification of representative sequences from each OTU was performed using the RDP classifier, with a minimal confidence of 60%. FastTree^52^ was then used to build a phylogenetic tree based on the aligned sequences. UniFrac-based beta-diversity was visualized after PCoA^53^. Over- or under-represented bacterial genera within a given category (diet, treatment, or diet-treatment combinations) were determined using the NSC method^54^. A heat map was made and hierarchical cluster analysis was performed by applying the Manhattan distance method to the processed and normalized data using PermutMatrix software^55^.

### Statistical analysis

Data were expressed as the mean ± SEM. All statistical analyses and Pearson’s correlation coefficients were performed using GraphPad Prism software (version 6.0; GraphPad Software, SD, USA). Pearson’s correlation coefficient heat maps were visualized using Excel and PowerPoint (version 2010; Microsoft Corporation, WA, USA). In experiments comparing multiple groups, differences were analysed by two-way ANOVA followed by Bonferroni’s post-hoc test. GTT and ITT were analysed using a repeated measure two-way ANOVA with both time and group as sources of variation. *P* values < 0.05 were regarded as significant (^*^P < 0.05 and ^**^P < 0.005).

## Additional Information

**How to cite this article**: Jung, M.-J. *et al*. Chronic Repression of mTOR Complex 2 Induces Changes in the Gut Microbiota of Diet-induced Obese Mice. *Sci. Rep.* **6**, 30887; doi: 10.1038/srep30887 (2016).

## Supplementary Material

Supplementary Information
