# Supplementary material for: Chronic Repression of mTOR Complex 2 Induces Changes in the Gut Microbiota of Diet-induced Obese Mice
Source: Sci Rep. 2016 Jul 29;6:30887. doi: 10.1038/srep30887 (PMC4965768; doi:10.1038/srep30887)
Supplement: Supplementary Information [file srep30887-s1.pdf]

SUPPLEMENTARY FIGURES AND TABLE

Chronic Repression of mTOR Complex 2 Induces Changes in the Gut Microbiota of Diet-induced Obese Mice

Mi-Ja Jung, Jina Lee, Na-Ri Shin, Min-Soo Kim, Dong-Wook Hyun, Ji-Hyun Yun, Pil Soo Kim, Tae Woong Whon, and Jin-Woo Bae

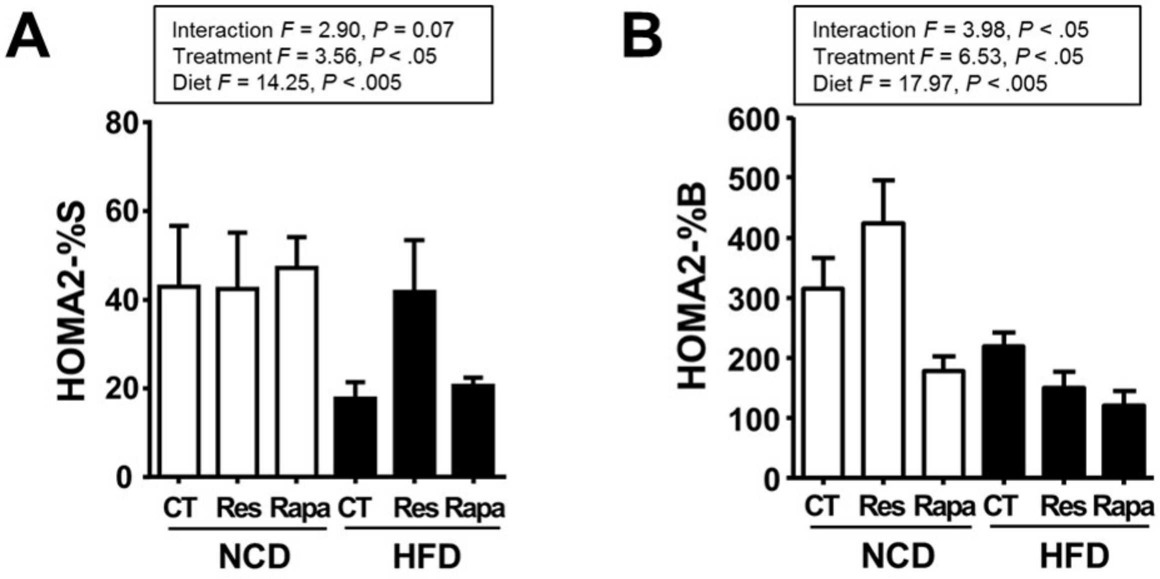

Supplementary Figure S1. Resveratrol improves, whereas rapamycin impairs, glucose homeostasis. (A) HOMA2-%S index and (B) QUICKI were calculated from fasting glucose and insulin levels (n = 5 per group). Data are expressed as the mean ± SEM. *F*- and *p*-values are from two-way ANOVA after Bonferroni's post hoc test. $^{*}P < .05$, $^{**}P < .005$.

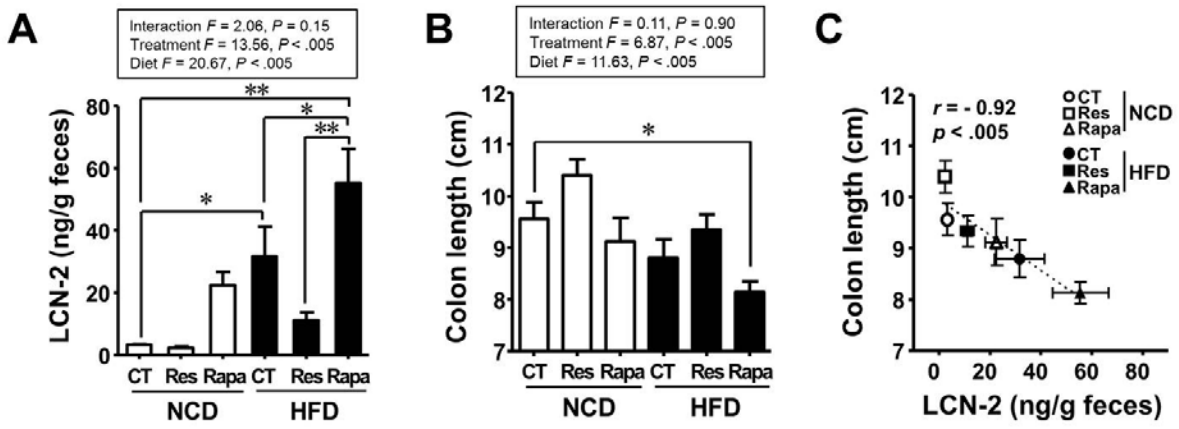

Supplementary Figure S2. Resveratrol ameliorates, whereas rapamycin exacerbates, intestinal inflammation. (A) The concentration of faecal LCN-2 (ng/g faeces) and (B) colon length (cm) were measured after 8 weeks of treatment (n = 5 per group). (C) The correlation between faecal LCN-2 concentrations and colon length was determined using two-tailed Pearson's *r* correlation analysis. Data are expressed as the mean ± SEM. *F*- and *p*-values are from two-way ANOVA after Bonferroni's post hoc test. $^{*}P < .05$, $^{**}P < .005$.

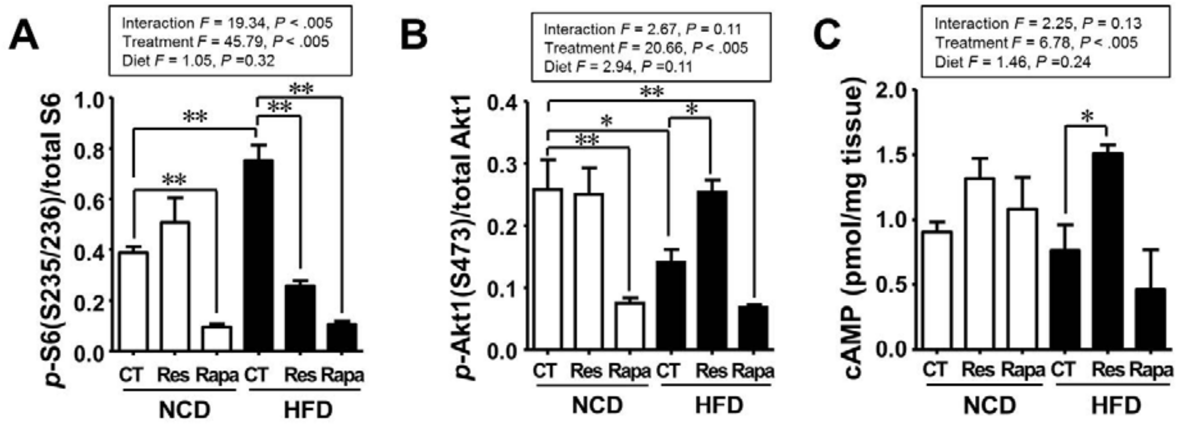

Supplementary Figure S3. Effects of resveratrol or rapamycin treatment on the mTOR signaling pathway in NCD- or HFD-fed mice. (A) p-S6 (Ser235/236)/total S6 (n = 5 per group), (B) p-Akt1 (Ser473)/total Akt1 (n = 3 per group) and (C) cAMP concentrations (n = 5 per group) in liver tissue samples. To examine Akt1 phosphorylation, mice were injected with 0.5U of insulin per kg body weight prior to sacrifice. Data are expressed as the mean ± SEM. *F*- and *p*-values are from two-way ANOVA after Bonferroni's post hoc test. $^{*}P < .05$, $^{**}P < .005$.

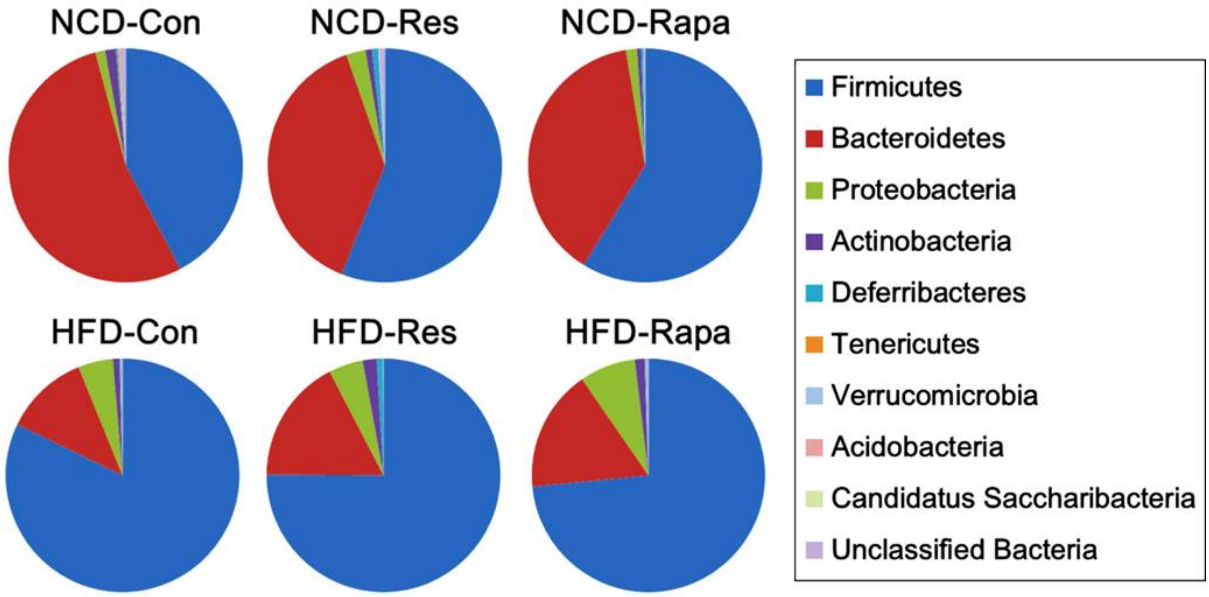

Supplementary Figure S4. Changes in the faecal bacterial community following resveratrol or rapamycin treatment. Pie charts showing the relative abundance (%) of different bacterial phyla in the different diet and treatment groups (n = 5 per group).

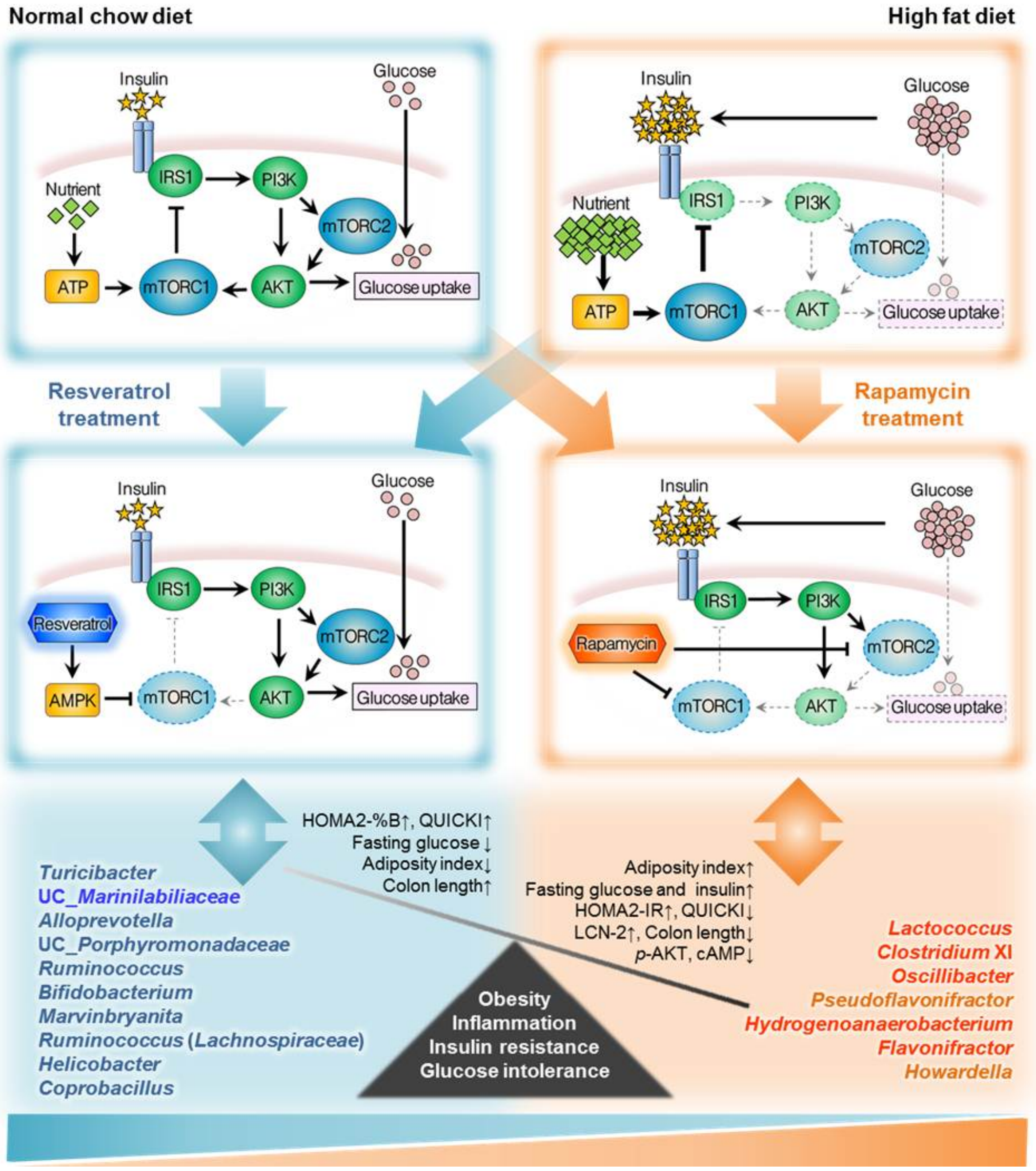

Supplementary Figure S5. Summary of the effects of resveratrol or rapamycin treatment on the interaction between host physiology and gut microbiota under normal-chow diet or high-fat diet conditions.

Supplementary Table S1. Alpha diversity of the gut microbial communities in NCD or HFD-fed mice after resveratrol or rapamycin treatment. 16S rRNA V1-2 sequences obtained by 454 pyrosquencing were used to analyze alpha diversity at the OTU level. *F*- and *p*-values are from two-way ANOVA after Bonferroni's post hoc test. $^{*}P < .05$; $^{**}P < .005$ compared with NCD-CT, $^{\#}P < .05$ compared with HFD-CT.

Group	# Observed OTUs	Chao1	Shannon	Phylogenetic Diversity	Simpson
NCD-CT	301±14	405±16	5.81±0.23	25.13±0.38	0.96±0.01
NCD-Res	306±24	432±30	5.90±0.11	24.86±0.85	0.96±0.01
NCD-Rapa	251±25	335±20	5.00±0.32	21.55±1.07*	0.92±0.01
HFD-CT	229±12*	318±29	5.07±0.13	20.49±0.82**	0.92±0.01
HFD-Res	171±6**	246±20**	4.48±0.06**	17.18±0.77**, #	0.91±0.01*
HFD-Rapa	215±19*	305±27*	5.15±0.37	18.89±1.21**	0.93±0.02
Interaction	F=3.72, P<.05	F=5.05, P<.05	F=6.06, P<.05	F=4.05, P<.05	F=5.14, P<.05
Treatment	F=1.69, P=0.21	F=1.39, P=0.27	F=1.36, P=0.28	F=4.44, P<.05	F=1.10, P=0.35
Diet	F=29.07, P<.005	F=24.60, P<.005	F=12.30, P<.005	F=47.12, P<.005	F=8.36, P<.05

Supplementary Table S2. *F*- and *p*-values of Figures 4A and 4B are from two-way ANOVA after Bonferroni's post hoc test.

Source of Variation	***Lactococcus***	***Clostridium XI***	***Oscillibacter***	***Pseudoflavonifractor***	***Flavonifractor***	***Hydrogeno-anaerobacterium***	***Howardella***
Interaction	$F=9.50$, $P<.005$	$F=17.61$, $P<.005$	$F=13.11$, $P<.005$	$F=23.30$, $P<.005$	$F=27.30$, $P<.005$	$F=10.98$, $P<.005$	$F=5.97$, $P<.05$
Treatment	$F=10.01$, $P<.005$	$F=15.84$, $P<.005$	$F=23.19$, $P<.005$	$F=11.71$, $P<.005$	$F=16.35$, $P<.005$	$F=3.88$, $P=0.07$	$F=8.92$, $P<.05$
Diet	$F=205.9$, $P<.005$	$F=85.69$, $P<.005$	$F=23.54$, $P<.005$	$F=13.51$, $P<.005$	$F=3.76$, $P=0.07$	$F=8.42$, $P<.05$	$F=26.27$, $P<.005$

Source of Variation	***Turicibacter***	***UC_Marinilabiliaceae***	***Alloprevotella***	***UC_Porphyro-monadaceae***	***Ruminococcus***	***Turicibacter***	***UC_Marinilabiliaceae***
Interaction	$F=17.61$, $P<.005$	$F=2.75$, $P=0.12$	$F=14.49$, $P<.005$	$F=7.37$, $P<.05$	$F=27.30$, $P<.005$	$F=10.53$, $P<.05$	$F=12.39$, $P<.005$
Treatment	$F=15.84$, $P<.005$	$F=3.0$, $P=0.10$	$F=19.87$, $P<.005$	$F=0.51$, $P=0.49$	$F=16.35$, $P<.005$	$F=10.44$, $P<.05$	$F=14.09$, $P<.005$
Diet	$F=85.69$, $P<.005$	$F=78.11$, $P<.005$	$F=20.84$, $P<.005$	$F=47.94$, $P<.005$	$F=3.76$, $P=0.07$	$F=10.50$, $P<.05$	$F=9.01$, $P<.05$

Source of Variation	***Alloprevotella***	***UC_Porphyro-monadaceae***	***Ruminococcus***
Interaction	$F=1.95$, $P=0.18$	$F=13.11$, $P<.005$	$F=3.36$, $P=0.09$
Treatment	$F=0.49$, $P=0.50$	$F=13.11$, $P<.005$	$F=2.84$, $P=0.11$
Diet	$F=21.40$, $P<.005$	$F=13.11$, $P<.005$	$F=7.60$, $P<.05$

Supplementary Table S3. Formula of normal chow diet (NCD).

Purina Lab. Rodent Chow (38057)					
Nutrients			***Minerals***		
**Protein**	**20**	**g%**	Ash	7.25	g%
Arginine	1.26	g%	Calcium	1.2	g%
Cystine	0.37	g%	Phosphorus	0.62	g%
Glycine	0.87	g%	Phosphorus (non-phytate)	0.4	g%
Histidine	0.5	g%	Potassium	0.82	g%
Isoleucine	0.82	g%	Magnesium	0.16	g%
Leucine	1.47	g%	Sulfur	0.22	g%
Lysine	1.01	g%	Sodium	0.34	g%
Methionine	0.33	g%	Chlorine	0.47	g%
Phenylalanine	0.98	g%	Fluorine	21.38	ppm
Tyrosine	0.63	g%	Iron	112.93	ppm
Threonine	0.72	g%	Zinc	128.85	ppm
Tryptophan	0.25	g%	Manganese	95.49	ppm
Valine	0.91	g%	Copper	22.74	ppm
			Cobalt	0.76	ppm
**Fat (ether extract)**	**4.5**	**g%**	Iodine	1.42	ppm
Linoleic Acid	1.1	g%	Chromium	0	ppm
Linolenic Acid	0.12	g%	Selenium	0.32	ppm
Arachidonic Acid	0.02	g%	***Vitamins***		
Omega-3 Fatty Acids	1.11	g%	Vitamin K	6.69	ppm
			Thiamin Hydrochloride	11.02	ppm
**Fiber (Crude)**	**3.7**	**g%**	Riboflavin	11.57	ppm
			Niacin	217.7	ppm
			Pantothenic Acid	88.72	ppm
			Choline Chloride	3447.96	ppm
			Folic Acid	13.6	ppm
			Pyridoxine	11	ppm
Calories provided by :			Biotin	0.15	ppm
**Protein**	**24.52**	%	B12	41	ppm
**Fat (ether extract)**	**12.41**	%	Vitamin A	28.03	IU/kg
**Carbohydrates**	**63.07**	%	Vitamin D3 (added)	4	IU/kg
**Total**	**3,940**	**kcal/kg**	Vitamin E	100	IU/kg

Supplementary Table S4. Formula of high-fat diet (HFD).

Research Diet #D12492	g%	kcal%
**Protein**	26.2	20
**Carbohydrate**	26.3	20
**Fat**	34.9	60
**Total**		100
**kcal/g**	5.24	
**Ingredient**	**g**	**kcal**
Casein, 30 Mesh	200	800
L-Cystine	3	12
Corn Starch	0	0
Maltodextrin 10	125	500
Sucrose	68.8	275.2
Cellulose, BW200	50	0
Soybean Oil	25	225
Lard*	245	2205
Mineral Mix S10026**	10	0
DiCalcium Phosphate	13	0
Calcium Carbonate	5.5	0
Potassium Citrate, 1 H2O	16.5	0
Vitamin Mix V10001***	10	40
Choline Bitartrate	2	0
FD&C Blue Dye #1	0.05	0
Total	773.85	4057

*Typical analysis of cholesterol in lard = 0.72 mg/g.
Cholesterol (mg)/4057 kcal = 216.4
Cholesterol (mg)/kg = 279.6

**Mineral Mix S10026	g
Sodium Chloride (39.3% Na 60.7% Cl)	2.59
Magnesium Oxide, Heavy, DC USP (60.3% Mg)	0.419
Magnesium Sulfate, Heptahydrate (9.87% Mg 13.0% S)	2.576
Ammonium Molybdate Tetrahydrate	0.003
Chromium Potassium Sulfate (10.4% Cr)	0.01925
Copper Carbonate (57.5% Cu)	0.0105
Ferric Citrate (17.4% Fe)	0.21
Manganese Carbonate Hydrate (47.8 Mn)	0.1225
Potassium Iodate (59.3% I)	0.00035
Sodium Fluoride (45.2%% Fl)	0.002
Sodium Selenite (45.7% Se)	0.00035
Zinc Carbonate (52.1% Zn)	0.056
Sucrose	3.99105

***Vitamin Mix V10001	g
Vitamin A Palmitate (500,000 IU/gm)	0.008
Vitamin D3 (100,000 IU/gm)	0.01
Vitamin E Acetate (500 IU/gm)	0.1
Menadione Sodium Bisulfite (62.5% Menadione)	0.0008
Biotin, 1.0%	0.02
Cyanocobalamin, 0.1%	0.01
Folic Acid	0.002
Nicotinic Acid	0.03
Calcium Pantothenate	0.016
Pyridoxine-HCl	0.007
Riboflavin	0.006
Thiamin HCl	0.006
Sucrose	9.78
